# Cyano­methyl 4-(4-methyl­benzene­sulfonamido)­benzoate

**DOI:** 10.1107/S1600536812017126

**Published:** 2012-04-21

**Authors:** Ghulam Mustafa, Mehmet Akkurt, Islam Ullah Khan, Tahir Muhmood

**Affiliations:** a Department of Chemistry, GC University, Lahore 54000, Pakistan; b Department of Physics, Faculty of Sciences, Erciyes University, 38039 Kayseri, Turkey

## Abstract

The title mol­ecule, C_16_H_14_N_2_O_4_S, adopts an L-shaped conformation, with the central C—S—N—C torsion angle being −69.1 (3)°. The two benzene rings form a dihedral angle of 89.94 (15)°. The mol­ecular conformation may be influenced by a weak intra­molecular C—H⋯O hydrogen bond which generates an *S*(6) ring motif. In the crystal, mol­ecules are linked by N—H⋯O and weak C—H⋯O hydrogen bonds, forming chains propagating along the *b* axis. Weak C—H⋯N hydrogen bonds connect the chains into a two-dimensional network parallel to (011). The crystal studied was an inversion twin, the ratio of components being 0.7 (1):0.3 (1).

## Related literature

For related structures, see: Mustafa *et al.* (2010 ▶, 2011 ▶, 2012*a* ▶,*b* ▶); Khan *et al.* (2011 ▶). For standard bond-length data, see: Allen *et al.* (1987 ▶). For hydrogen bond motifs, see: Bernstein *et al.* (1995 ▶).
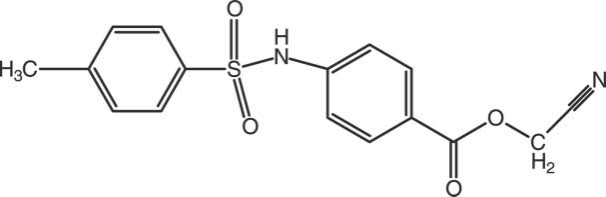


## Experimental

### 

#### Crystal data

C_16_H_14_N_2_O_4_S
*M* *_r_* = 330.36
Monoclinic, 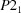

*a* = 5.9360 (3) Å
*b* = 8.1992 (4) Å
*c* = 15.9068 (8) Å
β = 91.222 (3)°
*V* = 774.02 (7) Å^3^
*Z* = 2
Mo *K*α radiation
μ = 0.23 mm^−1^
*T* = 296 K
0.28 × 0.23 × 0.19 mm

#### Data collection

Bruker APEXII CCD diffractometer
6266 measured reflections
3077 independent reflections
2306 reflections with *I* > 2σ(*I*)
*R*_int_ = 0.027

#### Refinement

*R*[*F* ^2^ > 2σ(*F* ^2^)] = 0.046
*wR*(*F* ^2^) = 0.106
*S* = 1.01
3077 reflections
210 parameters
1 restraint
H-atom parameters constrained
Δρ_max_ = 0.20 e Å^−3^
Δρ_min_ = −0.25 e Å^−3^
Absolute structure: Flack (1983 ▶), 1248 Freidel pairs
Flack parameter: 0.30 (10)

### 

Data collection: *APEX2* (Bruker, 2007 ▶); cell refinement: *SAINT* (Bruker, 2007 ▶); data reduction: *SAINT*; program(s) used to solve structure: *SHELXS97* (Sheldrick, 2008 ▶); program(s) used to refine structure: *SHELXL97* (Sheldrick, 2008 ▶); molecular graphics: *ORTEP-3 for Windows* (Farrugia, 1997 ▶) and *PLATON* (Spek, 2009 ▶); software used to prepare material for publication: *WinGX* (Farrugia, 1999 ▶) and *PLATON*.

## Supplementary Material

Crystal structure: contains datablock(s) global, I. DOI: 10.1107/S1600536812017126/lh5455sup1.cif

Structure factors: contains datablock(s) I. DOI: 10.1107/S1600536812017126/lh5455Isup2.hkl

Supplementary material file. DOI: 10.1107/S1600536812017126/lh5455Isup3.cml

Additional supplementary materials:  crystallographic information; 3D view; checkCIF report

## Figures and Tables

**Table 1:** Hydrogen-bond geometry (Å, °)

*D*—H⋯*A*	*D*—H	H⋯*A*	*D*⋯*A*	*D*—H⋯*A*
N1—H1⋯O3^i^	0.86	2.21	2.904 (3)	138
C1—H1*A*⋯O2^ii^	0.96	2.58	3.446 (5)	150
C9—H9⋯O2	0.93	2.38	3.025 (4)	126
C10—H10⋯O1^iii^	0.93	2.51	3.431 (4)	172
C12—H12⋯N2^iv^	0.93	2.62	3.426 (6)	146

Symmetry codes: (i) 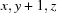; (ii) 
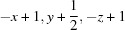
; (iii) 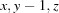; (iv) 
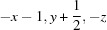
.

**Fig. 1.**
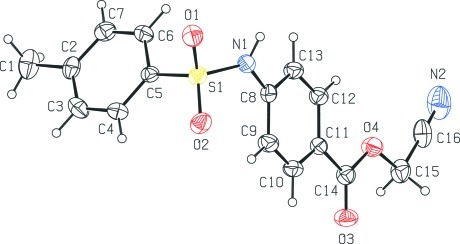
The molecular structure of (I) with displacement ellipsoids for non-H atoms drawn at the 30% probability level.

**Fig. 2.**
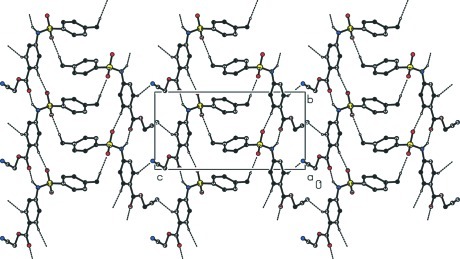
View of the packing and hydrogen-bonding interactions in (I) The hydrogen atoms not involved in the hydrogen bonds have been omitted.

## supplementary crystallographic information

### Comment

As part of our ongoing studies of sulfonamides with potential biological properties (Mustafa *et al.*, 2010, 2011, 2012*a*,*b*; Khan *et al.*, 2011), the crystal structure of the title compound (I) has been determined.

The molecular structure of (I) (Fig. 1), has a *L*-shaped conformation, with the central C5—S1—N1—C8 torsion angle being -69.1 (3)°. The two benzene rings (C2—C7) and (C8—C13) are nearly perpendicular to each other, with a dihedral angle of 89.94 (15)°. All the bond lengths (Allen *et al.*, 1987) and angles are normal (Mustafa *et al.*, 2010; 2011, 2012*a*,*b*; Khan *et al.*, 2011).

The title molecule exhibits an S(6) motif (Bernstein *et al.*, 1995) formed by a weak intramolecular C—H···O hydrogen bond interaction (Table 1). In the crystal, molecules are linked by N—H···O and weak C—H···O hydrogen bonds forming chains propagating along the *b* axis. Weak intermolecular C—H···N hydrogen bonds connect the chains into a two dimensional netwok (Table 1, Fig. 2).

### Experimental

To an aquious solution of *p*-amino benzoic acid (1.0 g, 7.3 mmol), sodium carbonate (1 N) was added to adjust the pH to 8. Then *p*-toluenesulfonyl chloride (1.80 g, 9.48 mmol) was added and the mixture was stirred at room temperature keeping the pH of the mixture at 8.0 with occasional addition of sodium carbonate solution. Progress and completion of the reaction was confirmed by TLC and conversion of the suspension into a clear solution. After 2 h, whole mixture was poured into a beaker and the pH was adjusted to 2.0 by 1 N HCl. Pprecipitates were produced which were filtered and washed with distilled water.

The prepared sulfonamide (4-(Toluene-4-sulfonylamino)-benzoic acid) (1.0 g, 3.43 mmol), DMF (10 ml) and n-hexane washed sodium hydride (0.25 g, 10.31 mmol) were stirred at room temperature for 40 min followed by the addition of chloroacetonitrile (0.34 g, 4.46 mmol). The whole reaction mixture was stirred at 353 K till the completion of the reaction and poured into crushed ice in a beaker. The pH of the mixture was adjusted to 4.0 with 1 N HCl. Precipitates were produced, filtered and washed twice with distilled water. Crystals suitable for X-ray diffraction were grown from a chloroform solution of the title compound.

### Refinement

All H atoms were positioned with idealized geometry and were refined using a riding model with *U*_iso_(H) = 1.2 or 1.5*U*_eq_(C) [N—H = 0.86 Å, C—H = 0.93, 0.96 or 0.97 Å]. One reflection (0 0 2) was omitted from the refinement. The crystal studied is an inversion twin with the refined BASF ratio of 0.70 (10)/0.30 (10).

### Crystal data

**Table:**

C_16_H_14_N_2_O_4_S	*F*(000) = 344
*M**_r_* = 330.36	*D*_x_ = 1.418 Mg m^−^^3^
Monoclinic, *P*2_1_	Mo *K*α radiation, λ = 0.71073 Å
Hall symbol: P 2yb	Cell parameters from 2045 reflections
*a* = 5.9360 (3) Å	θ = 2.6–24.7°
*b* = 8.1992 (4) Å	µ = 0.23 mm^−^^1^
*c* = 15.9068 (8) Å	*T* = 296 K
β = 91.222 (3)°	Block, yellow
*V* = 774.02 (7) Å^3^	0.28 × 0.23 × 0.19 mm
*Z* = 2	

### Data collection

**Table:**

Bruker APEXII CCD diffractometer	2306 reflections with *I* > 2σ(*I*)
Radiation source: sealed tube	*R*_int_ = 0.027
Graphite monochromator	θ_max_ = 27.1°, θ_min_ = 1.3°
φ and ω scans	*h* = −7→7
6266 measured reflections	*k* = −8→10
3077 independent reflections	*l* = −20→15

### Refinement

**Table:**

Refinement on *F*^2^	Secondary atom site location: difference Fourier map
Least-squares matrix: full	Hydrogen site location: inferred from neighbouring sites
*R*[*F*^2^ > 2σ(*F*^2^)] = 0.046	H-atom parameters constrained
*wR*(*F*^2^) = 0.106	*w* = 1/[σ^2^(*F*_o_^2^) + (0.0538*P*)^2^ + 0.0338*P*] where *P* = (*F*_o_^2^ + 2*F*_c_^2^)/3
*S* = 1.01	(Δ/σ)_max_ < 0.001
3077 reflections	Δρ_max_ = 0.20 e Å^−^^3^
210 parameters	Δρ_min_ = −0.25 e Å^−^^3^
1 restraint	Absolute structure: Flack (1983), 1248 Freidel pairs
Primary atom site location: structure-invariant direct methods	Flack parameter: 0.30 (10)

### Special details

**Table:**

Geometry. Bond distances, angles *etc*. have been calculated using the rounded fractional coordinates. All su's are estimated from the variances of the (full) variance-covariance matrix. The cell e.s.d.'s are taken into account in the estimation of distances, angles and torsion angles
Refinement. Refinement on *F*^2^ for ALL reflections except those flagged by the user for potential systematic errors. Weighted *R*-factors *wR* and all goodnesses of fit *S* are based on *F*^2^, conventional *R*-factors *R* are based on *F*, with *F* set to zero for negative *F*^2^. The observed criterion of *F*^2^ > σ(*F*^2^) is used only for calculating -*R*-factor-obs *etc*. and is not relevant to the choice of reflections for refinement. *R*-factors based on *F*^2^ are statistically about twice as large as those based on *F*, and *R*-factors based on ALL data will be even larger.

### Fractional atomic coordinates and isotropic or equivalent isotropic displacement parameters (Å^2^)

**Table:**

	*x*	*y*	*z*	*U*_iso_*/*U*_eq_	
S1	0.58175 (11)	0.32017 (11)	0.30134 (5)	0.0453 (2)	
O1	0.6354 (4)	0.4862 (3)	0.28356 (14)	0.0570 (8)	
O2	0.7581 (3)	0.2041 (3)	0.31249 (15)	0.0597 (9)	
O3	0.1129 (4)	−0.4816 (3)	0.16303 (18)	0.0630 (10)	
O4	−0.1469 (4)	−0.3362 (3)	0.09421 (15)	0.0576 (8)	
N1	0.4185 (4)	0.2648 (3)	0.22249 (17)	0.0505 (9)	
N2	−0.6118 (7)	−0.3907 (7)	−0.0148 (3)	0.1056 (19)	
C1	0.0250 (6)	0.3400 (6)	0.6140 (2)	0.0774 (14)	
C2	0.1617 (5)	0.3314 (5)	0.53585 (18)	0.0512 (10)	
C3	0.3637 (6)	0.2464 (4)	0.5345 (2)	0.0595 (12)	
C4	0.4901 (5)	0.2381 (4)	0.4627 (2)	0.0515 (11)	
C5	0.4149 (4)	0.3166 (4)	0.39131 (16)	0.0400 (8)	
C6	0.2138 (5)	0.4021 (4)	0.3910 (2)	0.0487 (11)	
C7	0.0899 (5)	0.4073 (4)	0.4624 (2)	0.0542 (11)	
C8	0.3266 (5)	0.1077 (4)	0.20785 (18)	0.0422 (10)	
C9	0.4346 (5)	−0.0359 (4)	0.2306 (2)	0.0538 (11)	
C10	0.3380 (4)	−0.1827 (5)	0.20995 (18)	0.0508 (9)	
C11	0.1348 (4)	−0.1913 (5)	0.16607 (16)	0.0416 (8)	
C12	0.0272 (5)	−0.0468 (4)	0.1442 (2)	0.0466 (11)	
C13	0.1211 (5)	0.0997 (4)	0.1656 (2)	0.0484 (11)	
C14	0.0402 (5)	−0.3518 (4)	0.1432 (2)	0.0481 (11)	
C15	−0.2527 (6)	−0.4841 (4)	0.0670 (2)	0.0621 (12)	
C16	−0.4543 (8)	−0.4347 (5)	0.0209 (3)	0.0704 (17)	
H1	0.38520	0.33880	0.18600	0.0610*	
H1A	0.02430	0.45000	0.63460	0.1160*	
H1B	0.08990	0.26940	0.65610	0.1160*	
H1C	−0.12670	0.30610	0.60120	0.1160*	
H3	0.41510	0.19380	0.58310	0.0710*	
H4	0.62470	0.18000	0.46270	0.0620*	
H6	0.16340	0.45560	0.34250	0.0590*	
H7	−0.04630	0.46340	0.46160	0.0650*	
H9	0.57210	−0.03270	0.25960	0.0640*	
H10	0.41080	−0.27870	0.22580	0.0610*	
H12	−0.10980	−0.04980	0.11480	0.0560*	
H13	0.04560	0.19560	0.15150	0.0580*	
H15A	−0.29080	−0.55120	0.11490	0.0740*	
H15B	−0.15360	−0.54570	0.03110	0.0740*	

### Atomic displacement parameters (Å^2^)

**Table:**

	*U*^11^	*U*^22^	*U*^33^	*U*^12^	*U*^13^	*U*^23^
S1	0.0447 (3)	0.0424 (4)	0.0486 (4)	−0.0002 (4)	−0.0010 (3)	−0.0054 (4)
O1	0.0656 (14)	0.0481 (15)	0.0572 (15)	−0.0175 (12)	0.0007 (11)	0.0017 (11)
O2	0.0456 (12)	0.0650 (16)	0.0684 (17)	0.0109 (12)	−0.0035 (12)	−0.0136 (13)
O3	0.0728 (17)	0.0376 (14)	0.078 (2)	0.0009 (12)	−0.0101 (15)	0.0073 (13)
O4	0.0626 (13)	0.0432 (13)	0.0662 (16)	−0.0060 (11)	−0.0166 (11)	−0.0016 (12)
N1	0.0686 (16)	0.0394 (17)	0.0430 (16)	−0.0007 (12)	−0.0077 (13)	−0.0001 (11)
N2	0.080 (3)	0.147 (4)	0.089 (3)	−0.016 (3)	−0.017 (2)	−0.030 (3)
C1	0.081 (2)	0.087 (3)	0.065 (2)	−0.032 (3)	0.0211 (18)	−0.016 (3)
C2	0.0556 (16)	0.0490 (19)	0.0493 (18)	−0.0153 (19)	0.0056 (13)	−0.012 (2)
C3	0.069 (2)	0.060 (2)	0.049 (2)	−0.0071 (17)	−0.0092 (17)	0.0152 (16)
C4	0.0490 (16)	0.0492 (19)	0.056 (2)	0.0085 (15)	−0.0042 (16)	0.0071 (16)
C5	0.0401 (12)	0.0322 (13)	0.0473 (15)	0.0002 (17)	−0.0056 (11)	−0.0017 (18)
C6	0.0481 (17)	0.0507 (18)	0.047 (2)	0.0090 (16)	−0.0053 (15)	0.0056 (15)
C7	0.0441 (16)	0.0535 (19)	0.065 (2)	0.0053 (16)	0.0008 (16)	−0.0030 (18)
C8	0.0480 (16)	0.0437 (19)	0.0352 (17)	0.0022 (15)	0.0048 (13)	−0.0013 (14)
C9	0.0486 (17)	0.051 (2)	0.061 (2)	0.0068 (16)	−0.0147 (16)	−0.0020 (17)
C10	0.0517 (14)	0.0448 (17)	0.0554 (18)	0.011 (2)	−0.0074 (13)	0.002 (2)
C11	0.0458 (12)	0.0407 (16)	0.0383 (15)	0.0018 (18)	0.0024 (11)	0.0028 (17)
C12	0.0435 (17)	0.047 (2)	0.049 (2)	0.0035 (15)	−0.0040 (15)	−0.0004 (16)
C13	0.0458 (17)	0.044 (2)	0.055 (2)	0.0056 (16)	−0.0082 (17)	−0.0011 (18)
C14	0.0471 (17)	0.048 (2)	0.049 (2)	−0.0018 (17)	−0.0003 (16)	−0.0013 (18)
C15	0.072 (2)	0.057 (2)	0.057 (2)	−0.0181 (19)	−0.0040 (19)	−0.0070 (18)
C16	0.066 (3)	0.095 (3)	0.050 (3)	−0.018 (2)	−0.0030 (19)	−0.015 (2)

### Geometric parameters (Å, º)

**Table:**

S1—O1	1.428 (3)	C9—C10	1.370 (5)
S1—O2	1.423 (2)	C10—C11	1.382 (3)
S1—N1	1.633 (3)	C11—C12	1.387 (5)
S1—C5	1.758 (3)	C11—C14	1.473 (5)
O3—C14	1.189 (4)	C12—C13	1.364 (5)
O4—C14	1.349 (4)	C15—C16	1.448 (6)
O4—C15	1.429 (4)	C1—H1A	0.9600
N1—C8	1.416 (4)	C1—H1B	0.9600
N2—C16	1.142 (7)	C1—H1C	0.9600
N1—H1	0.8600	C3—H3	0.9300
C1—C2	1.501 (4)	C4—H4	0.9300
C2—C7	1.383 (4)	C6—H6	0.9300
C2—C3	1.388 (5)	C7—H7	0.9300
C3—C4	1.382 (5)	C9—H9	0.9300
C4—C5	1.372 (4)	C10—H10	0.9300
C5—C6	1.384 (4)	C12—H12	0.9300
C6—C7	1.367 (4)	C13—H13	0.9300
C8—C9	1.385 (5)	C15—H15A	0.9700
C8—C13	1.382 (4)	C15—H15B	0.9700
			
O1—S1—O2	119.72 (14)	O3—C14—O4	121.9 (3)
O1—S1—N1	104.13 (14)	O4—C14—C11	111.3 (3)
O1—S1—C5	108.00 (15)	O4—C15—C16	105.6 (3)
O2—S1—N1	109.43 (14)	N2—C16—C15	177.8 (5)
O2—S1—C5	108.25 (14)	C2—C1—H1A	109.00
N1—S1—C5	106.57 (13)	C2—C1—H1B	109.00
C14—O4—C15	116.5 (3)	C2—C1—H1C	109.00
S1—N1—C8	126.8 (2)	H1A—C1—H1B	109.00
S1—N1—H1	117.00	H1A—C1—H1C	109.00
C8—N1—H1	117.00	H1B—C1—H1C	110.00
C3—C2—C7	117.6 (3)	C2—C3—H3	119.00
C1—C2—C3	121.3 (3)	C4—C3—H3	119.00
C1—C2—C7	121.1 (3)	C3—C4—H4	120.00
C2—C3—C4	121.5 (3)	C5—C4—H4	120.00
C3—C4—C5	119.2 (3)	C5—C6—H6	120.00
S1—C5—C6	119.3 (2)	C7—C6—H6	120.00
S1—C5—C4	120.2 (2)	C2—C7—H7	119.00
C4—C5—C6	120.4 (3)	C6—C7—H7	119.00
C5—C6—C7	119.5 (3)	C8—C9—H9	120.00
C2—C7—C6	121.8 (3)	C10—C9—H9	120.00
C9—C8—C13	119.1 (3)	C9—C10—H10	119.00
N1—C8—C13	117.1 (3)	C11—C10—H10	119.00
N1—C8—C9	123.8 (3)	C11—C12—H12	120.00
C8—C9—C10	119.7 (3)	C13—C12—H12	120.00
C9—C10—C11	121.5 (3)	C8—C13—H13	120.00
C12—C11—C14	122.0 (2)	C12—C13—H13	119.00
C10—C11—C12	118.4 (3)	O4—C15—H15A	111.00
C10—C11—C14	119.6 (3)	O4—C15—H15B	111.00
C11—C12—C13	120.4 (3)	C16—C15—H15A	111.00
C8—C13—C12	121.0 (3)	C16—C15—H15B	111.00
O3—C14—C11	126.9 (3)	H15A—C15—H15B	109.00
			
O1—S1—N1—C8	176.9 (2)	C3—C4—C5—C6	−0.5 (5)
O2—S1—N1—C8	47.8 (3)	C3—C4—C5—S1	175.5 (3)
C5—S1—N1—C8	−69.1 (3)	C4—C5—C6—C7	−0.2 (5)
O1—S1—C5—C4	−118.0 (3)	S1—C5—C6—C7	−176.3 (2)
O2—S1—C5—C4	13.0 (3)	C5—C6—C7—C2	1.1 (5)
N1—S1—C5—C4	130.6 (3)	N1—C8—C13—C12	175.8 (3)
O1—S1—C5—C6	58.1 (3)	C9—C8—C13—C12	−1.8 (5)
O2—S1—C5—C6	−171.0 (2)	N1—C8—C9—C10	−176.6 (3)
N1—S1—C5—C6	−53.3 (3)	C13—C8—C9—C10	0.9 (4)
C15—O4—C14—C11	179.3 (2)	C8—C9—C10—C11	0.5 (4)
C14—O4—C15—C16	176.3 (3)	C9—C10—C11—C12	−1.0 (4)
C15—O4—C14—O3	−0.3 (4)	C9—C10—C11—C14	178.3 (3)
S1—N1—C8—C9	−32.5 (4)	C10—C11—C14—O4	−174.3 (2)
S1—N1—C8—C13	150.0 (2)	C12—C11—C14—O3	−175.5 (3)
C1—C2—C7—C6	179.3 (3)	C12—C11—C14—O4	5.0 (4)
C1—C2—C3—C4	180.0 (3)	C10—C11—C14—O3	5.2 (5)
C3—C2—C7—C6	−1.1 (5)	C10—C11—C12—C13	0.1 (4)
C7—C2—C3—C4	0.3 (5)	C14—C11—C12—C13	−179.2 (3)
C2—C3—C4—C5	0.5 (5)	C11—C12—C13—C8	1.3 (5)

### Hydrogen-bond geometry (Å, º)

**Table:**

*D*—H···*A*	*D*—H	H···*A*	*D*···*A*	*D*—H···*A*
N1—H1···O3^i^	0.86	2.21	2.904 (3)	138
C1—H1*A*···O2^ii^	0.96	2.58	3.446 (5)	150
C9—H9···O2	0.93	2.38	3.025 (4)	126
C10—H10···O1^iii^	0.93	2.51	3.431 (4)	172
C12—H12···N2^iv^	0.93	2.62	3.426 (6)	146

Symmetry codes: (i) *x*, *y*+1, *z*; (ii) −*x*+1, *y*+1/2, −*z*+1; (iii) *x*, *y*−1, *z*; (iv) −*x*−1, *y*+1/2, −*z*.
